# Feasibility of Reducing the Fiber Content in Ultra-High-Performance Fiber-Reinforced Concrete under Flexure

**DOI:** 10.3390/ma10020118

**Published:** 2017-01-28

**Authors:** Jung-Jun Park, Doo-Yeol Yoo, Gi-Joon Park, Sung-Wook Kim

**Affiliations:** 1 Structural Engineering Research Institute, Korea Institute of Civil Engineering and Building Technology, 283 Daehwa-dong, Goyangdae-ro, Ilsanseo-gu, Goyang-si, Gyeonggi-do 10223, Korea; jjpark@kict.re.kr (J.-J.P); joon7767@kict.re.kr (G.-J.P); swkim@kict.re.kr (S.-W.K); 2 Department of Architectural Engineering, Hanyang University, 222 Wangsimni-ro, Seongdong-gu, Seoul 04763, Korea

**Keywords:** ultra-high-performance fiber-reinforced concrete, flexure, toughness, low fiber contents, fiber length

## Abstract

In this study, the flexural behavior of ultra-high-performance fiber-reinforced concrete (UHPFRC) is examined as a function of fiber length and volume fraction. Straight steel fiber with three different lengths (*l_f_*) of 13, 19.5, and 30 mm and four different volume fractions (*v_f_*) of 0.5%, 1.0%, 1.5%, and 2.0% are considered. Test results show that post-cracking flexural properties of UHPFRC, such as flexural strength, deflection capacity, toughness, and cracking behavior, improve with increasing fiber length and volume fraction, while first-cracking properties are not significantly influenced by fiber length and volume fraction. A 0.5 vol % reduction of steel fiber content relative to commercial UHPFRC can be achieved without deterioration of flexural performance by replacing short fibers (*l_f_* of 13 mm) with longer fibers (*l_f_* of 19.5 mm and 30 mm).

## 1. Introduction

The brittleness and low strength-to-weight ratio of ordinary concrete are critical drawbacks limiting its practical application in structures subjected to tension or flexure. To reduce brittleness, the addition of discontinuous fiber, such as steel fiber, polymeric fiber, and carbon fiber, has been widely investigated [1,2,3]. This method is both simple and efficient. Randomly oriented fibers at a crack surface resist the external tensile load through fiber bridging, leading to increased toughness. Furthermore, the strength-to-weight ratio can be improved by lowering the water-to-cementitious material ratio, as the increase in weight is small compared to the increase in strength.

However, the increase in strength of conventional fiber-reinforced concrete (FRC) is limited by fiber breakage before complete pullout [4], especially when deformed fibers are inclined in direction of pullout load. In the mid-1990s, ultra-high-performance fiber-reinforced concrete (UHPFRC), exhibiting both excellent strength (compressive strength ≥ 150 MPa and tensile strength ≥ 8 MPa [5]) and toughness, was successfully developed [6]. An ultra-high-strength cement matrix was adopted, and a large amount of high strength non-deformed (straight) micro steel fiber (fiber volume fraction (*v_f_*) = 2.0%) was incorporated into the ultra-high-strength cement matrix to prevent breakage. Since the early 2000s, both the material and structural properties of UHPFRC have been actively investigated [7,8,9,10,11,12,13,14,15].

Despite its excellent mechanical properties, the practical application of UHPFRC has been limited due to its high manufacturing price. In particular, the price of high-strength steel fiber accounts for about 33% of the total manufacturing cost [14]. Therefore, decreasing the amount of steel fiber without sacrificing tensile or flexural performance is a key challenge remaining to be solved before widespread adoption of UHPFRC can be realized. Wille et al. [13] reported a UHPFRC with a relatively high tensile strength and ductility, made using deformed (end-hooked or twisted) fibers at a low fiber volume fraction. They [13] found that the post-cracking strain capacity (ε*_pc_* = 0.6%) of a UHPFRC with 1.5 vol % of twisted steel fibers is almost double that of conventional UHPFRC with short straight steel fibers. However, to fabricate the deformed steel fiber, an additional manufacturing process is required (to deform the fiber), increasing both manufacturing time and cost. Yoo et al. [7,8] proposed a simpler way to improve the flexural performance of UHPFRC by using longer straight steel fiber. Experiments [8,9] showed improvement of uniaxial and biaxial flexural performance of commercial UHPFRC after replacing short fibers with long fibers while keeping the volume fraction constant (*v_f_* = 2.0%). However, their studies [8,9] focused on improving the flexural performance of UHPFRC by changing the fiber length; based on their results alone, it is not straightforward to quantitatively determine how much increasing the fiber length will allow the volume fraction to be reduced in order to maintain the same performance.

This study investigates the flexural properties of UHPFRC with several fiber lengths and volume fractions. Three straight steel fiber lengths and four volume fractions are considered. The specific objectives are: (1) to evaluate the effects of fiber length and volume fraction on the flexural properties of UHPFRC in terms of strength, deflection capacity, toughness, and cracking behavior; and (2) to quantitatively estimate how much the fiber volume fraction can be decreased from the commercial UHPFRC by replacing short fibers with long fibers.

## 2. Experimental Program

### 2.1. Materials, Mixture Proportions, and Specimen Preparation

Type I Portland cement and silica fume were used as cementitious materials. Their chemical compositions and physical properties are summarized in Table 1. Silica sand with a grain size between 0.2 and 0.3 mm was used as a fine aggregate, and silica flour with a grain size of about 10 μm and a composition of 98% SiO_2_ was used as filler. Coarse aggregate was not included in this study to improve flexural performance, similar to ordinary UHPFRC used by many researchers [6,7,8,16]. 1.6% (by cement weight) superplasticizer, a high-range water reducing agent, was also applied to provide proper fluidity. Detailed mixture proportions are given in Table 2.

**Figure 1:**
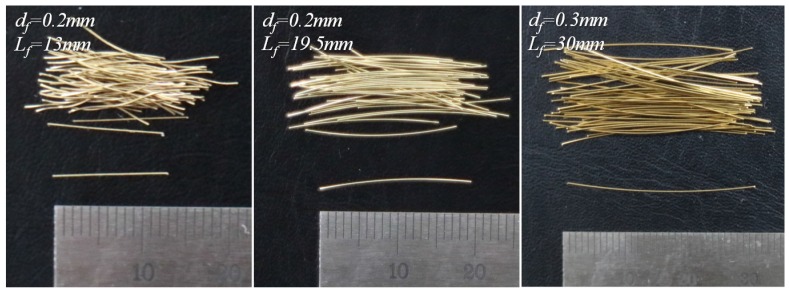
Picture for straight steel fibers.

In order to investigate the effect of increasing the fiber length and fiber content on the flexural performance of UHPFRC, three types of straight steel fibers with lengths of 13, 19.5, and 30 mm (short, medium, and long) and four different fiber volume fractions (*v_f_*) of 0.5%, 1.0%, 1.5%, and 2.0% have been studied. Geometrical and physical properties are summarized in Table 3 and shown in Figure 1. For the long straight steel fibers (L), a larger diameter of 0.3 mm was used to prevent fiber breakage before complete pullout [9].

Since the composition of UHPFRC is different from ordinary concrete, a unique mixing sequence is required. First, all of the dry components such as cement, silica fume, silica sand, and silica flour are included in the mixer and premixed for about 10 min to achieve good dispersion. Water and superplasticizer are then added to the dry components and mixed for another 10 min. Once the mixture has adequate fluidity and viscosity, steel fibers are carefully dispersed and mixed for another 5 min.

The flexural performance of UHPFRC is significantly influenced by the concrete placement method [9,17]. Therefore, concrete was consistently placed parallel to the longitudinal direction in all of the tested beams in order to provide uniform fiber orientation and dispersion. All of the fibers satisfied the ASTM C1609 [18] requirements for the width and depth of test specimens (100 × 100 mm^2^), which should be three times larger than the maximum fiber length.

### 2.2. Compressive Test

A total of 36 cylindrical specimens (three cylinders for each test variable, i.e., each combination of fiber length and volume fraction) with a diameter of 100 mm and a height of 200 mm were used. The casting surface of all cylinders was properly ground using a diamond blade before testing in order to exclude the eccentric loading effect. A uniaxial load was applied through a universal testing machine (UTM) with a maximum load capacity of 2500 kN at a rate of 0.1 mm/min (stroke speed).

### 2.3. Four-Point Flexural Test (ASTM C1609)

**Figure 2:**
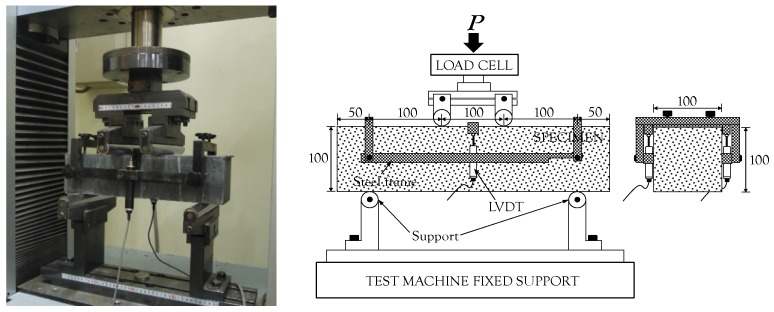
Four-point flexural test as per ASTM C1609 [18].

Four prismatic beams with dimensions 100 × 100 × 400 mm^3^ were fabricated and tested for each test variable. Test methods and procedures were followed as specified by ASTM C1609 [18]. A uniaxial load was applied through a UTM with a maximum load capacity of 250 kN at a rate of 0.4 mm/min (stroke speed). The clear span length was 300 mm, and a pin-type support system was incorporated at both sides. To measure the mid-span deflection without a support settlement, a steel frame with two linear variable differential transformers (LVDTs) was installed at the middle height of the beam. A load cell included in the cross head was also used for measuring the applied load. The detailed setup for the four-point flexural tests is shown in Figure 2. In order to examine multiple micro-cracking behavior, the bottom surface of the beam was sprayed with three layers of polyurethane before testing.

## 3. Experimental Results and Discussion

### 3.1. Compressive Strength

**Figure 3:**
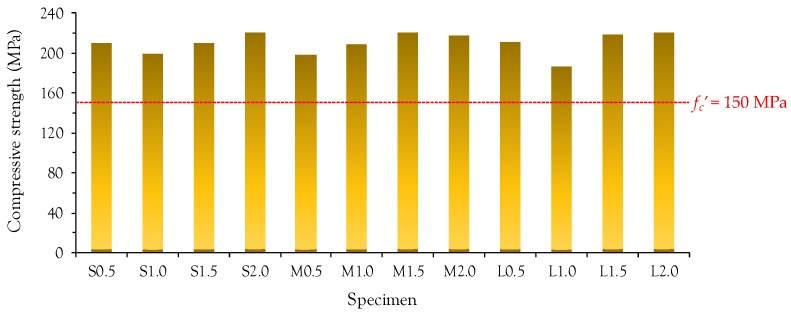
Summary of compressive strength.

Figure 3 summarizes the average compressive strengths for all test series. There is no obvious trend with fiber length and volume fraction. All of the tested cylinders exhibited compressive strengths larger than 150 MPa, which is the minimum strength required by AFGC-SETRA specifications [5]. Due to its high homogeneity, all of the UHPFRC cylinders showed a linear compressive stress versus strain relationship and failed with a sudden drop in the compressive stress immediately after reaching the peak point, indicating a brittle failure mode. No fragmentation was observed in any of the specimens due to the fiber bridging effect.

### 3.2. Flexural Load Versus Deflection Behaviors

**Figure 4:**
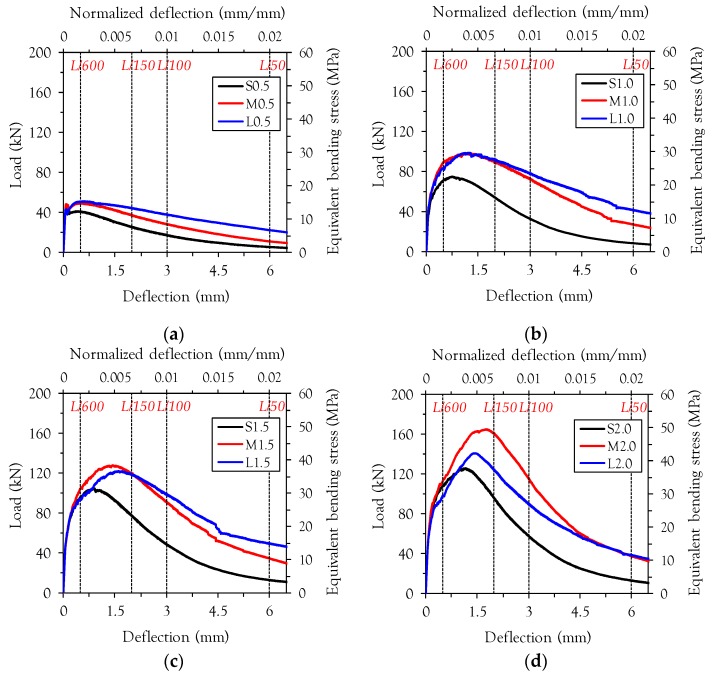
Average load versus deflection curves; (**a**) *v_f_* = 0.5%; (**b**) *v_f_* = 1.0%; (**c**) *v_f_* = 1.5%; (**d**) *v_f_* = 2.0%

Figure 4 shows the average flexural load versus deflection (or equivalently, bending stress versus normalized deflection) curves for all test series. In most cases, test results obtained from four beams were used for obtaining the average curve. However, only three beams were used for the case of specimen M1 with *v_f_* of 2%, because the test data from one specimen was not recorded due to technical problem. In Appendix, all test results are also given to provide information regarding deviation of the test data. Yoo et al. [9] reported that the first cracking point, called the limit of proportionality (LOP), can be clearly determined from the flexural load and deflection (or crack mouth opening displacement) relationship for UHPFRC with 2 vol % steel fiber. Accordingly, the first cracking point in this study is referred to as the LOP. Most of the UHPFRC exhibits deflection-hardening behavior, showing a higher load carrying capacity after the first cracking until a second peak is reached; this post-cracking peak point is referred to as the modulus of rupture (MOR).

UHPFRC beams with short straight steel fiber (the S-series) exhibited the worst flexural performance with regard to the flexural strength (or called the equivalently bending strength) and energy absorption capacity (or called the toughness), regardless of the fiber volume content. The specimen S0.5, with a *v_f_* of 0.5%, exhibited deflection-softening behavior (*f*_LOP_ > *f*_MOR_), whereas the specimens M0.5 and L0.5 showed deflection-hardening behavior (*f*_LOP_ < *f*_MOR_), where *f*_LOP_ is the first-cracking flexural strength and *f*_MOR_ is the post-cracking flexural strength. For *v_f_* higher than 1.0%, all of the UHPFRC beams exhibited deflection-hardening behavior regardless of the fiber type. Interestingly, similar or slightly better performance in terms of flexural strength and post-peak ductility was obtained in the specimen with long fibers (L-series) than the specimen with medium length fiber (M-series) at low fiber volume fractions up to a *v_f_* of 1.5%. On the other hand, the specimen with medium length steel fiber exhibited the best flexural behavior at the highest volume fraction of 2.0%. For example, the *f*_MOR_ of M2.0 was found to be 49.5 MPa, which is approximately 31% higher than S2.0 and 17% higher than L2.0. The most likely explanation for this observation is that fiber alignment in the direction of the tensile load is disturbed in the specimen with long fibers due to increased fiber-fiber interactions at high volume fractions (*v_f_* of 2.0%), compared to the specimen with medium length fibers. Martinie and Roussel [19] found that fiber orientation is significantly influenced by fiber-fiber interactions at high volume fractions.

### 3.3. Flexural Properties at the Points of LOP and MOR

**Figure 5:**
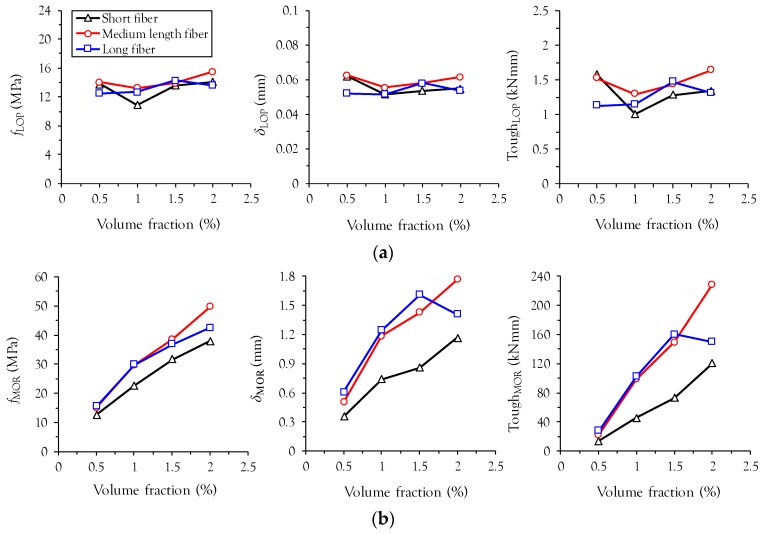
Effect of fiber length and volume fraction on strength, deflection capacity, and toughness; (**a**) at limit of proportionality (LOP); (**b**) at modulus of Rupture (MOR).

Table 4 summarizes the properties of UHPFRC beams tested as per ASTM C1609 [18]. As shown in Figure 5, the first-cracking properties (*f*_LOP_, δ_LOP_, and Tough_LOP_) are not significantly influenced by fiber length and volume fraction, consistent with findings from previous studies [10]. This is mainly because the first-cracking properties are more closely related to matrix tensile cracking than fiber bridging capacity. Alternatively, the post-cracking properties at the MOR are strongly influenced by fiber length and volume fraction: (1) higher values of *f*_MOR_, δ_MOR_, and Tough_MOR_ were obtained with an increase in the fiber volume fraction and (2) specimens with medium length and long fibers exhibited higher values of *f*_MOR_, δ_MOR_, and Tough_MOR_ than specimens with short fibers. Values of *f*_MOR_ increased almost linearly with volume fraction in the specimens with short and medium length fibers, whereas a more gradual increase was obtained in the specimen with long fibers above a volume fraction of 1.0%. In particular, a lower deflection capacity was observed in L2.0 than in L1.5, resulting in a lower value of Tough_MOR_ at a *v_f_* of 2.0%. Up to a *v_f_* of 1.5%, similar values of *f*_MOR_, δ_MOR_, and Tough_MOR_ are obtained in specimens with medium length and long fibers.

**Figure 6:**
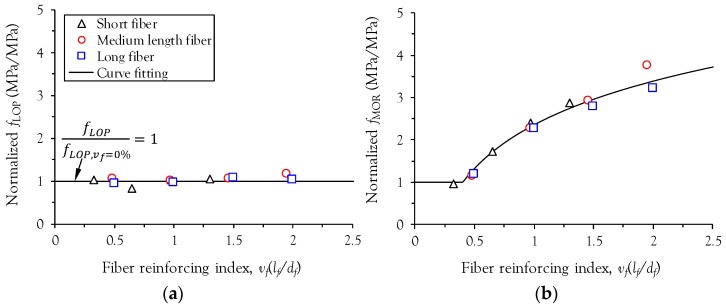
Relationship between normalized strengths and fiber reinforcing index of ultra-high-performance fiber-reinforced concrete (UHPFRC); (**a**) first-cracking strength *f_LOP_*; (**b**) post-cracking strength *f_MOR_.*

To investigate the effect of the fiber reinforcing index, *v_f_* (*l_f_*/*d_f_*) on the normalized *f*_LOP_ and *f*_MOR_ (*f*_LOP_/*f*_LOP_, *v_f_* = 0% and *f*_MOR_/*f*_LOP_, *v_f_* = 0%) of UHPFRC, the first-cracking strength of plain ultra-high-performance concrete (UHPC) without fiber, *f*_LOP_, *v_f_* = 0%, was measured as per ASTM C1609 [18] and found to be 13.2 MPa. Since plain UHPC has no second peak point after matrix cracking, the first-cracking strength, *f*_LOP_, *v_f_* = 0%, indicates the modulus of rupture, *f*_MOR_, *v_f_* = 0%, as well. Therefore, both *f*_LOP_ and *f*_MOR_ in UHPFRC are normalized to the identical value of *f*_LOP_, *v_f_* = 0%. Figure 6 shows the relationship between the normalized strength and the fiber reinforcing index of UHPFRC with various steel fibers. It is obvious that the normalized *f*_LOP_ improves slightly with a larger fiber reinforcing index, but the improvement is relatively minor compared with the normalized *f*_MOR_. The minor effect of the fiber volume fraction on the first-cracking tensile strength of UHPFRC was also numerically verified by Yoo et al. [11]. On the other hand, the normalized *f*_MOR_ clearly increases with the fiber reinforcing index (Figure 6b). The relationship between the normalized *f*_MOR_ and the fiber reinforcing index is not significantly influenced by the length of straight steel fibers. If the fiber reinforcing index, *v_f_* (*l_f_*/*d_f_*), is lower than approximately 0.4, deflection-softening behavior will be observed. For this case, the flexural strength, *f*_MOR_, of UHPFRC should be identical to the first-cracking strength, *f*_LOP_, and thus the normalized *f*_MOR_ is equal to 1, as shown in Figure 6b.

### 3.4. Energy Absorption Capacity (Toughness)

In order to investigate the energy absorption capacity, four mid-span deflection points were adopted in this study as follows:

– L/600: at the point where the mid-span deflection is 0.5 mm
– L/150: at the point where the mid-span deflection is 2 mm
– L/100: at the point where the mid-span deflection is 3 mm
– L/50: at the point where the mid-span deflection is 6 mm

ASTM C1609 [18] recommends evaluating the toughness of FRC at the deflection points of L/600 and L/150. On the other hand, since UHPFRC exhibits excellent load carrying capacity even at large deflections, two additional points at L/100 and L/50 were also considered.

**Figure 7:**
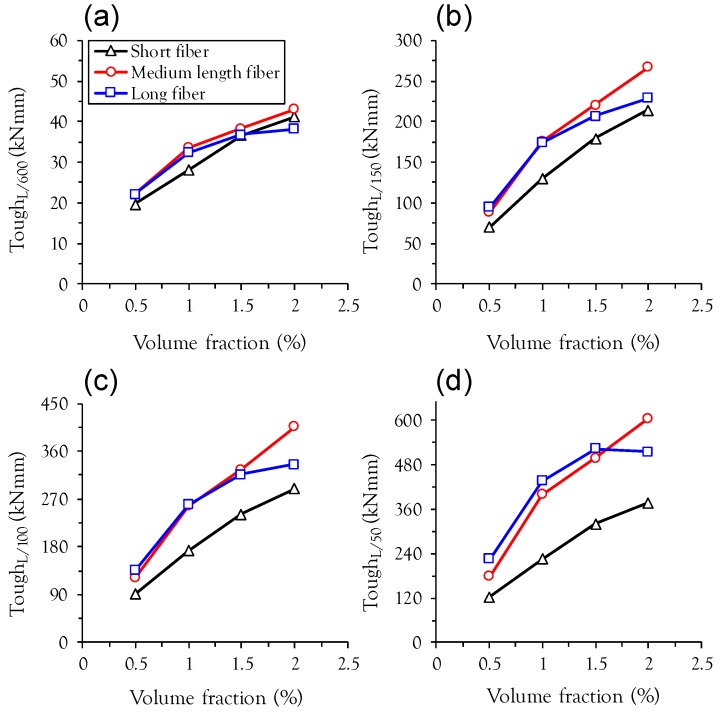
Effect of fiber length and volume fraction on energy absorption capacity at; (**a**) L/600; (**b**) L/150; (**c**) *L*/100; (**d**) L/50.

**Figure 8:**
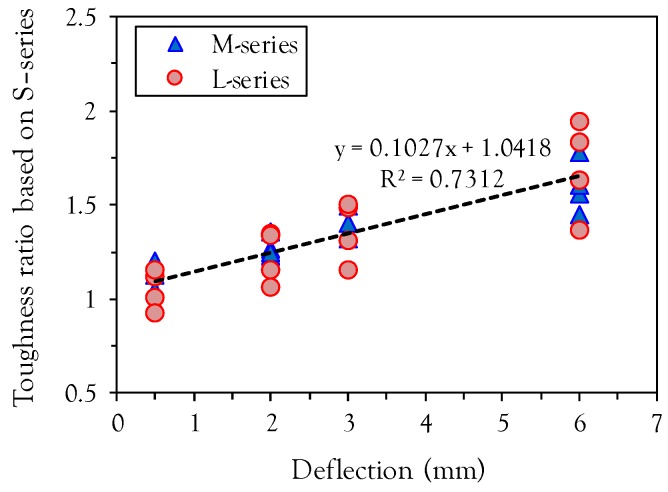
Relationship between toughness ratio based on S-series and mid-span deflection.

The effects of fiber length and volume content on the energy absorption capacity at various mid-span deflection points are summarized in Figure 7. Regardless of fiber length and deflection points, the toughness increases with the fiber volume fraction due to improved post-cracking flexural properties such as flexural strength, deflection capacity, and post-peak ductility. At the lower mid-span deflection point (L/600), similar values of toughness were obtained in all test specimens. However, the difference between the toughnesses of specimens with short fibers (S-series) and medium length or long fibers (M- or L-series) increases with increasing mid-span deflection points. This is also supported by Figure 8, which shows the relationship between the toughness ratio relative to the specimen with short fiber and the mid-span deflections. For both medium length and long fibers, the toughness ratio increases with increasing mid-span deflection, because longer fibers with more bonding area between the fiber and matrix can sustain a greater tensile load at larger crack opening displacements than shorter fibers. For example, the toughness of L1.0 at the 6 mm deflection point was found to be 436.5 kN·mm, approximately 94% higher than S1.0 (224.7 kN·mm) at the identical deflection point.

### 3.5. Cracking Behaviors

**Figure 9:**
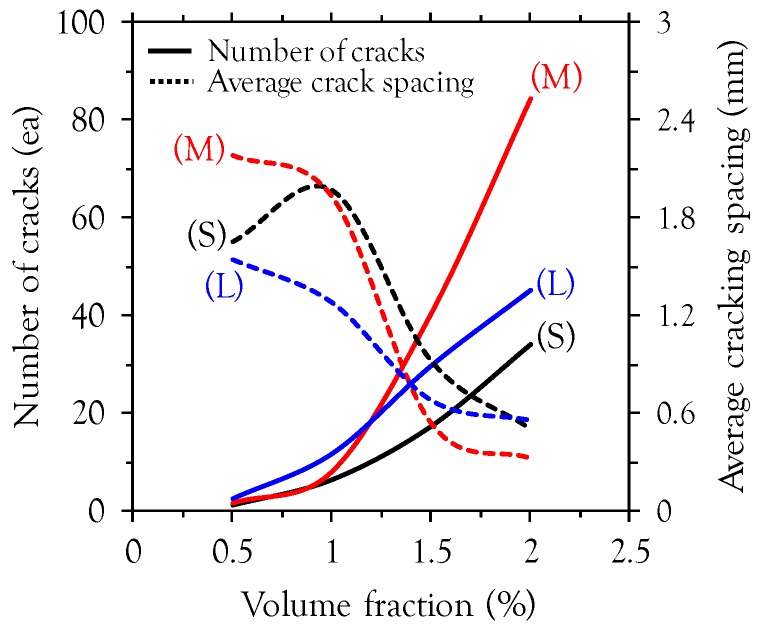
Cracking behaviors (S = short fiber, M = medium length fiber, and L = long fiber).

**Figure 10:**
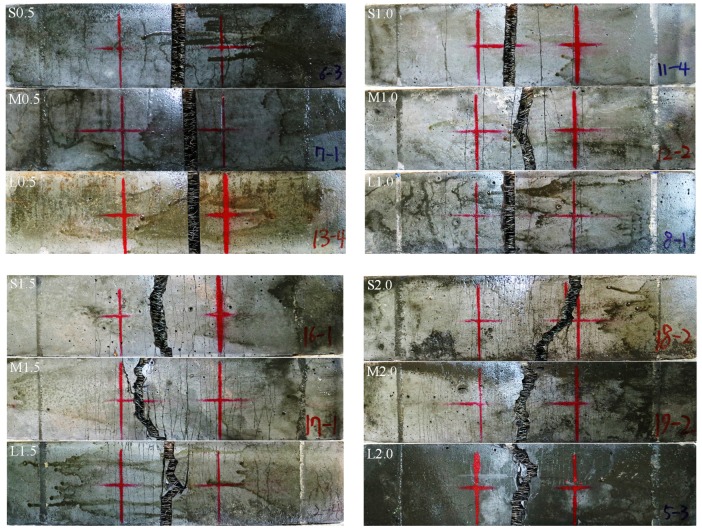
Picture for cracking patterns.

The effects of fiber length and volume fraction on the cracking behavior, including the number of cracks and average crack spacings, are shown in Figure 9. To detect very fine cracks with the naked eye, alcohol was sprayed onto the bottom surface after testing. The alcohol percolated into the micro-cracks so that they could be clearly detected, as shown in Figure 10. Increasing the volume fraction caused the number of cracks to increase and the average crack spacing to decrease. This indicates that more micro-cracks are formed between earlier cracks at higher fiber volume fractions. Interestingly, the best cracking behavior in terms of the number of cracks and average crack spacing was obtained in specimens with long fibers at low fiber volume fractions (*v_f_* ≤ 1.0%), while at high fiber volume fractions (*v_f_* > 1.5%) the best cracking behavior was obtained in specimens with medium length fibers. This is consistent with the findings of the flexural properties at MOR in Figure 5b. All of the tested beams exhibited crack localization where the width of one specific crack increased among multiple micro-cracks after reaching the point of MOR (Figure 10).

### 3.6. Effectiveness of Increasing the Fiber Length on Reducing the Fiber Contents in Commercial Uhpfrc without Degradation of Flexural Performance

**Figure 11:**
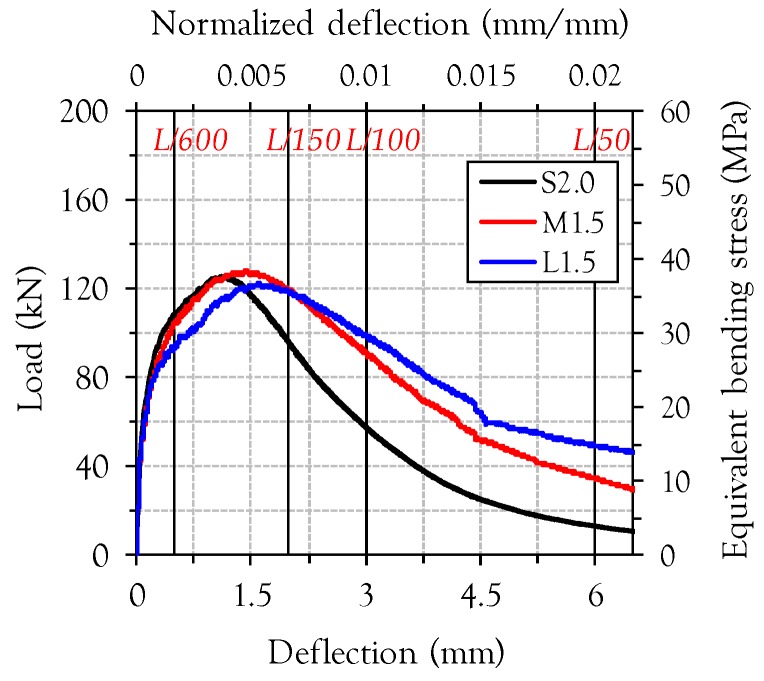
Comparison of flexural behaviors between commercial UHPFRC (S2.0) and M1.5 (or L1.5).

**Figure 12:**
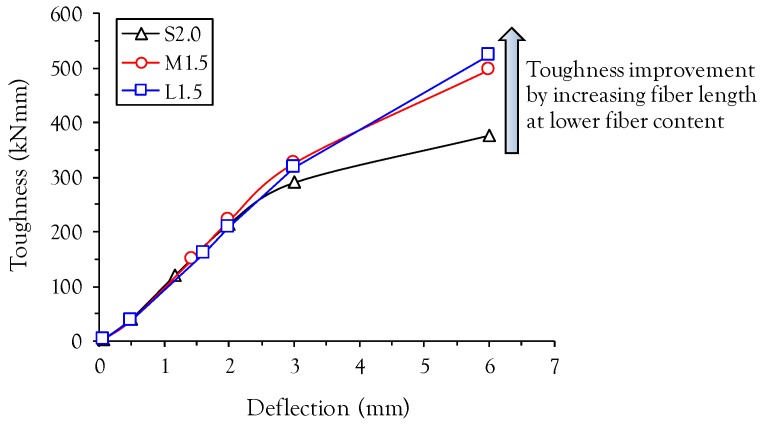
Comparison of toughnesses between commercial UHPFRC (S2.0) and M1.5 (or L1.5).

Figure 11 shows a comparison between the flexural behaviors of the specimen with 2 vol % short fibers (S2.0), commercially available UHPFRC in North America [7], and specimens with 1.5 vol % medium length and long fibers (M1.5 and L1.5). As can be seen in Figure 11, specimens M1.5 and L1.5 exhibited similar flexural strength and higher deflection capacity and post-peak ductility (a more gradual decrease in the load carrying capacity versus deflection after the peak) compared to specimen S2.0. Specimens M1.5 and L1.5 even exhibited higher toughness values at mid-span deflections larger than 2 mm than specimen S2.0, as shown in Figure 12. A possible explanation for this observation is that there is a higher possibility of fibers existing at crack surfaces for longer fibers (M- and L-series) than for shorter fibers (S-series), so that a similar bonding area between the fiber and the matrix is obtained in specimens S2.0 and M1.5 (or L1.5) even though the actual amount of fibers included in M1.5 and L1.5 is substantially lower. Yoo and Banthia [20] recently reported that the difference between the number of fibers per unit area detected at the crack surface in specimens with short and long fibers was relatively smaller than that between the actual numbers of fibers included in the mixtures, owing to the higher possibility of fibers existing at a random crack surface for the long fibers than for the short fibers. Therefore, we conclude that the volume fraction of steel fibers can be reduced by approximately 0.5% simply by replacing short fibers (*l_f_*/*d_f_* = 13/0.2 = 65) with medium length (*l_f_*/*d_f_* = 19.5/0.2 = 97.5) or long (*l_f_*/*d_f_* = 30/0.3 = 100) fibers from commercially available UHPFRC (S2.0), with a corresponding improvement in the energy absorption capacity.

## 4. Conclusions

This study investigated the flexural behavior of UHPFRC with various lengths and volume fractions of straight steel fibers. A way to decrease the fiber content from the commercial UHPFRC without degradation of the flexural performance is suggested. From the above discussion, the following conclusions can be drawn:

1. The flexural performance of UHPFRC with short straight steel fibers can be improved by increasing the fiber length. The positive effect of using long fibers on the flexural performance is diminished at high fiber volume fractions (*v_f_* of 2.0%).
2. At low fiber volume fractions (*v_f_* ≤ 1.0%), the best cracking response was obtained in UHPFRC with long fibers, whereas at high fiber volume fractions (*v_f_* > 1.5%), the best cracking response was observed in that with medium length fibers.
3. The normalized *f*_LOP_ was not influenced by the fiber reinforcing index, whereas the normalized *f*_MOR_ obviously increases with the fiber reinforcing index.
4. Toughness improves with increasing fiber length and volume fraction. The effectiveness of using longer fibers on improving toughness was most pronounced at larger deflections.
5. By replacing short fibers with medium length or long fibers, the volume fraction of steel fibers in commercial UHPFRC can be reduced by approximately 0.5% without any deterioration of flexural strength, along with a slight improvement in the energy absorption capacity.

## Figures and Tables

**Table 1:** Chemical compositions and physical properties of cementitious materials.

Composition % (mass)	Type I Portland Cement	Silica Fume
CaO	61.33	0.38
Al_2_O_3_	6.40	0.25
SiO_2_	21.01	96.00
Fe_2_O_3_	3.12	0.12
MgO	3.02	0.10
SO_3_	2.30	-
Specific Surface Area (cm^2^/g)	3413	200,000
Density (g/cm^3^)	3.15	2.10

**Table 2:** Mixture proportions.

W/B	Unit Weight (kg/m^3^)					
	Water	Cement	Silica Fume	Silica Sand	Silica Flour	Superplasticizer
0.2	160.3	788.5	197.1	867.4	236.6	52.6

W/B = water-to-binder ratio.

**Table 3:** Geometrical and physical properties of steel fibers.

Name	*d_f_* (mm)	*l_f_* (mm)	Aspect Ratio (*l_f_*/*d_f_*)	Density (g/cm^3^)	*f_t_* (MPa)	*E_f_* (GPa)
S M L	0.2 0.2 0.3	13.0 19.5 30.0	65.0 97.5 100.0	7.9 7.9 7.9	2788 2500 2580	200 200 200

*d_f_* = fiber diameter, *l_f_* = fiber length, *f_t_* = tensile strength of fiber, and *E_f_* = elastic modulus of fiber.

**Table 4:** Summary of properties obtained from flexural tests ASTM C1609 [18]).

Parameters		Unit	S0.5	S1.0	S1.5	S2.0	M0.5	M1.0	M1.5	M2.0	L0.5	L1.0	L1.5	L2.0
LOP	*P*_LOP_	kN	45.9	36.3	45.3	47.0	46.8	44.2	46.3	51.8	41.7	42.3	47.6	45.3
	*f*_LOP_	MPa	13.8	10.9	13.6	14.1	14.0	13.3	13.9	15.5	12.5	12.7	14.3	13.6
	δ_LOP_	mm	0.062	0.052	0.054	0.055	0.063	0.056	0.058	0.062	0.052	0.052	0.058	0.054
	Tough_LOP_	kN·mm	1.57	1.01	1.28	1.34	1.53	1.29	1.43	1.64	1.13	1.15	1.47	1.31
L/600	*P*_L/600_	kN	40.8	71.6	95.7	108.4	49.6	90.0	104.9	113.5	51.1	83.4	94.3	97.9
	*f*_L/600_	MPa	12.3	21.5	28.7	32.5	14.9	27.0	31.5	34.0	15.3	25.0	28.3	29.4
	δ_L/600_	mm	0.5	0.5	0.5	0.5	0.5	0.5	0.5	0.5	0.5	0.5	0.5	0.5
	Tough_L/600_	kN·mm	19.81	28.05	36.65	41.21	22.23	33.66	38.29	43.03	22.22	32.47	36.75	38.22
MOR	*P*_MOR_	kN	41.5	75.2	104.9	125.9	49.6	98.8	128.0	165.1	51.5	99.0	122.2	141.1
	*f*_MOR_	MPa	12.4	22.6	31.5	37.8	14.9	29.6	38.4	49.5	15.4	29.7	36.7	42.3
	δ_MOR_	mm	0.36	0.74	0.86	1.17	0.51	1.18	1.43	1.76	0.61	1.25	1.61	1.41
	Tough_MOR_	kN·mm	13.86	45.75	73.07	120.59	22.38	98.36	149.25	228.27	27.80	102.22	160.20	149.80
L/150	*P*_L/150_	kN	25.8	54.1	77.5	95.9	37.3	89.8	119.6	161.0	44.6	91.8	118.9	124.3
	*f*_L/150_	MPa	7.74	16.2	23.2	28.8	11.2	26.9	35.9	48.3	13.4	27.5	35.7	37.3
	δ_L/150_	mm	2.0	2.0	2.0	2.0	2.0	2.0	2.0	2.0	2.0	2.0	2.0	2.0
	Tough_L/150_	kN·mm	70.38	129.75	179.70	214.81	88.73	176.01	220.52	267.04	94.60	174.09	207.18	228.95
L/100	*P*_L/100_	kN	17.3	33.1	48.7	57.9	28.6	72.8	91.0	115.8	38.2	78.4	99.4	90.6
	*f*_L/100_	MPa	5.19	9.93	14.61	17.38	8.58	21.85	27.31	34.74	11.45	23.5	29.8	27.2
	δ_L/100_	mm	3.0	3.0	3.0	3.0	3.0	3.0	3.0	3.0	3.0	3.0	3.0	3.0
	Tough_L/100_	kN·mm	91.78	172.69	242.01	290.17	121.49	257.53	326.06	406.07	135.91	259.35	316.57	355.23
L/50	*P*_L/50_	kN	5.7	8.8	13.1	13.1	11.4	27.4	34.6	37.7	22.5	41.8	49.5	38.6
	*f*_L/50_	MPa	1.70	2.63	3.94	3.94	3.42	8.21	10.38	11.32	6.76	12.5	14.8	11.6
	δ_L/50_	mm	6.0	6.0	6.0	6.0	6.0	6.0	6.0	6.0	6.0	6.0	6.0	6.0
	Tough_L/50_	kN·mm	123.04	224.66	320.03	370.94	178.49	398.68	497.42	605.63	225.85	436.48	523.09	514.63

*P* = flexural load, *f* = flexural stress, δ = mid-span deflection, Tough = toughness, LOP = limit of proportionality, MOR = modulus of rupture, L/600 = δ of 0.5 mm, L/150 = δ of 2 mm, L/100 = δ of 3 mm, and L/50 = δ of 6 mm.

**Figure A1:**
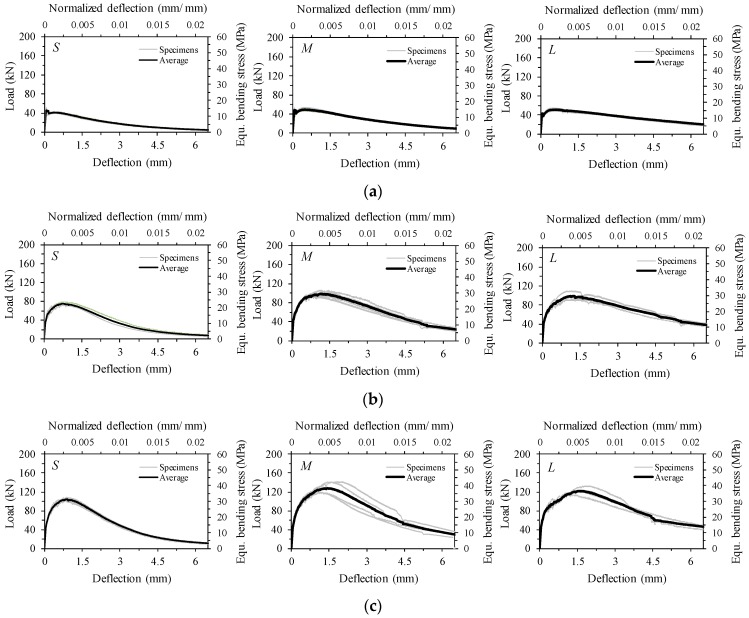

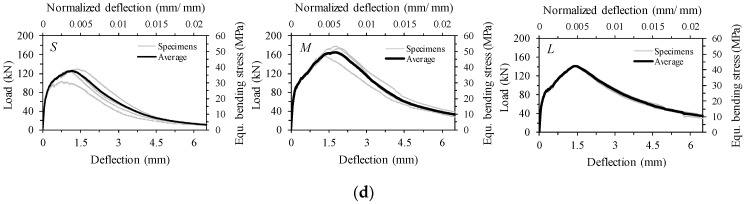
Summary of load versus deflection curves; (**a**) *v_f_* = 0.5%, (**b**) *v_f_* = 1.0%, (**c**) *v_f_* = 1.5%, (**d**) *v_f_* = 2.0%.

## Appendix A

Summary of flexural load versus deflection curves of all test specimens is given in Figure A1. The *S*, *M*, and *L* indicate short, medium-length, and long fibers. Average curve was obtained based on a linear interpolation method with an equal increase of deflection in 0.5 μm. So, the data point in *y*-axis (load) is equally spaced according to the *x*-axis (deflection).
